# Antibiotic Resistance: Challenges and Strategies in Combating Infections

**DOI:** 10.7759/cureus.46013

**Published:** 2023-09-26

**Authors:** Jay Chavada, Komal N Muneshwar, Yash Ghulaxe, Mohit Wani, Prayas P Sarda, Shreyash Huse

**Affiliations:** 1 Department of Community Medicine, Jawaharlal Nehru Medical College, Datta Meghe Institute of Higher Education and Research, Wardha, IND

**Keywords:** enzyme inhibitor, antibiotic resistance, anti-microbial resistance, conventional antibiotics, combined modality therapy

## Abstract

From a broader perspective, antibiotic or antimicrobial resistance is still evolving and spreading internationally. Infectious diseases have become more complex and often impossible to cure, increasing morbidity and mortality. Despite the failure of conventional, standard antimicrobial therapy, no new class of antibiotics has been developed in the last 20 years, which results in various cutting-edge and other tactics that can be used to encounter these disease-causing microorganisms with antibiotic resistance. In the continued fight against bacterial infections, there is an urgent requirement for new antibiotics and other antimicrobials. Antibiotic resistance is inevitable, and pharmaceutical companies consistently show little interest in funding novel antibiotic research. Some methods are being used as a possible replacement for conventional antibiotics. Combination therapy, methods that target the proteins or enzymes that cause antimicrobial resistance and bacterial resistance, systems for delivery of the drug, physicochemical approaches, and informal ways, such as the CRISPR-Cas system, are some of these approaches. These various approaches influence how multi-drug-resistant organisms are handled in human clinical settings.

## Introduction and background

Antibiotics are used for both the prevention and cure of bacterial infections. Continuous use of antibiotics makes bacteria resist, leading to antibiotic resistance (AR). A scenario when bacteria change and stop responding to medication increases the risk of spread of disease, serious diseases, and death and makes diseases challenging to treat [1]. When the capacity of bacteria to resist the outcome of antibiotics at therapeutically feasible concentrations increases, it is referred to as bacterial resistance [2]. The growing global issue known as AR is due to the lack of ability of medicine to manage the infectious diseases for which they were designed. Now that the World Health Organization (WHO) has warned that the world is "running out of antibiotics," and that the concerns about the rise of AR worldwide are intensifying. There has recently been a significant challenge in treating clinical infectious disorders due to the growth of antibiotic-resistant bacteria, which has led to a rise in the prevalence of nosocomial infections [3]. Because infections due to bacteria are the leading causes of illness and mortality, AR is increasing worldwide. The recurring emergence of new resistance mechanisms and their global dissemination threaten the ability to cure common infectious diseases. Resistance to antibiotics is a severe healthcare problem due to its potential for global spread, and limited treatment choices would arise [4]. Infections like pneumonia, TB, gonorrhea, and food-borne disorders become more challenging; it is impossible to cure the disease when antibiotics are ineffective. Infections due to bacteria are now dangerous, decades after the first patients got antibiotic therapy. If prompt and proactive action is not taken, we are quickly moving into a post-antibiotic world in which routine diseases and small wounds could be fatal again. AR was recently listed as one of the top 10 worldwide public health dangers to humanity by the WHO [1].

## Review

Resistance to antibiotics and the rise in drug-resistant bacteria

Sir Alexander Fleming predicted the risks of incorrect penicillin usage and the onset of resistance in his acceptance speech for the Nobel Prize in 1945 [5]. Antibiotics that are effective for treating general bacterial infections, including sepsis, hospital-acquired infections, sexually transmitted infections, urinary tract infections (UTI), and diarrhea, are becoming increasingly scarce globally [2]. The resistance rates for *Klebsiella pneumoniae* and *Escherichia coli* to the antibiotic ciprofloxacin, which is frequently used to treat UTI, were 8.4% to 92.9% and 4.1% to 79.4%, respectively, according to the WHO information sheet on antimicrobial resistance [1]. *Klebsiella pneumoniae* can produce fatal infections, and carbapenem AR is an issue affecting the entire world. The following factors all have an impact on the intricate and varied issue of AR: a) Poor adherence to septic precautions and hospital etiquette, which contribute to a rise in AR in bacteria, and excessive antibiotic usage in agriculture are two factors that contribute to the spread of resistance. b) Rise in improper healthcare facility hygiene. c) Trade and international travel, which can cause the spreading of resistant germs. d) Poor sanitation in some locations can pollute water supplies and spread bacteria that are resistant to treatment. e) A lack of fast diagnosis, among other things, prevents proper use of antibiotics [6,7].

Bacterial resistance strategies against antibiotics

To flourish in the presence of an antibiotic, the bacterial strains must be able to try to hinder a few of the crucial stages required for the antimicrobial drug to function well. Species of bacteria use any of four fundamental survival techniques: a) Reducing the antibiotic's capacity to enter the microbial cell, preventing it from reaching the antibiotic's target in the bacteria. b) The efflux pump mechanism helps the cell remove antibacterial drugs. c) Modification or breakdown results in antibiotic deactivation. d) Modifying or altering the bacteria's antibacterial target [8].

Epidemiology of AR

Around the world, AR is a leading reason of death and a financial burden. Incidences of several serious multi-drug-resistant (MDR) bacterial infections have increased since 2013, according to the US Centers for Disease Control and Prevention, including an erythromycin-resistant group A Streptococcus infection that has increased by 315%, a drug-resistant *Neisseria gonorrhoea* infection that has increased by 124%, and an extended-spectrum lactamase-producing Enterobacteriaceae infection that has increased by 50% [9]. Similarly, there was a 3.5% increase in the prevalence of resistance to vancomycin *Streptococcus aureus* between 2006 and 2020, with Africa recording the most significant percentage (16%) [10]. According to a WHO report, multi-drug-resistant tuberculosis (MDR-TB) has infected an estimated 3.3% of recent tuberculosis (TB) cases and 18% of those already treated. A serious risk is the development of resistance to recent "last resort" TB medications used for the treatment of drug-resistant TB. Low-income and middle-income nations are highly affected because of widespread misuse of antibiotics, use of antibiotics in agriculture, inappropriate drug quality, inadequate supervision, and some other factors linked to subpar standards of health care, malnutrition, recurrent and chronic infections, and the lack of ability to afford more potent and expensive medications [11].

Problems militating the development of new antibiotics

As existing medications and drug classes lose their efficacy, new drugs and drug classes must constantly be developed to maintain the use of antibiotics in managing infectious diseases. The demand for novel anti-infective medications in health care is immense, and time is rapidly exhausting. Although there is a continued need for innovative antimicrobial medicines, high-yield pharmaceutical companies have dropped this idea. The companies have moved out of the research and development of antibacterial drugs because of the increased expenditure on clinical trials, advanced regulatory ambiguity around licensing needs, and a low return [12,13]. This decline can be attributed to many factors, such as regulatory need that requires lengthened and demanding clinical trials for validation of antibiotics as well as the low economic return in financing for antibiotics in comparison to some different therapeutics, the difficulty of exploring newer compounds using traditional discovery methods, the belief that resistance will arise with no doubt to new antimicrobials, and other factors [14].

Emerging strategies for tackling AR

There is an urgent need for answers to the persistent danger of AR, as well as the associated risks, illness, mortality, and economic losses. Despite the fact that many new antibiotics are now being created, according to data published by the WHO, they are still anticipated to be effective in combating the most significant forms of bacterial resistance against antibiotics [1].

Immune Antibiotics as an Alternative

Historically, antibiotics' direct repressing and killing effects are the only biological activities considered [15]. Contrarily, it is becoming more and more apparent that antimicrobials collaborate along the host's native immunity to provide notable indirect effects that enhance the clearance of bacteria, which may result in faster and more significant effects, reducing the likelihood of resistance emergence within residual bacteria [15]. According to several findings, native endogenous host defense peptides have developed to save the host by preventing pathogenic organisms from causing infection and have antimicrobial modes of action similar to peptide antimicrobials administered to patients such as colistin and daptomycin [16]. Undoubtedly, scientific study has been done on the synergistic, additive, neutralizing, and antagonistic effects of antibiotics, but little is known about how antibiotics interact with the innate immune system [17,18]. Although an antibiotic's direct in vitro mode of action is believed to make it effective against bacteria, extra antibiotic effects on the bacteria have been connected to improve immune optimization and host immunological activities [19]. This incorporates how antibiotics work with other immune systems and virulence factors to influence response to the host. In a study by Volk et al., it was found that patients with methicillin-resistant *Staphylococcus aureus* (MRSA) bacteremia treated with lactam adjunctive therapy in combination with both conventional antibiotics produced less IL-10 and more IL-1 as a result of the influence of antibiotic responses on the immune system of the host [20]. Even while earlier attempts to generate a *Streptococcus aureus* vaccine were unsuccessful, newly developed immunologic-based medicines that incorporate anti-virulence factor antibodies with conventional medications seem to promise [21].

Biochemical Methods to Reduce Resistance and Increase Susceptibility to Currently Available Antibiotics

**Figure 1:**
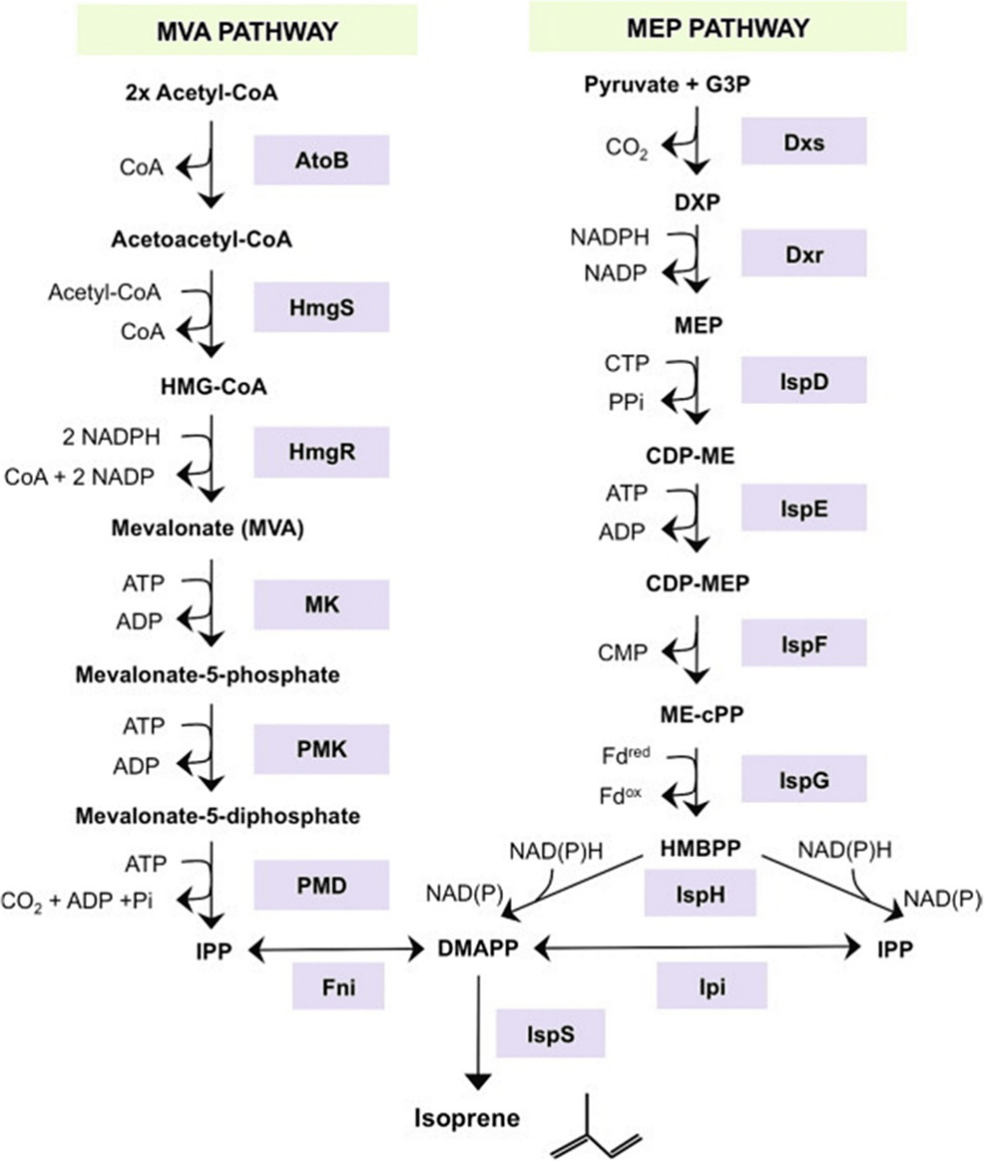
Mevalonate (MVA) methyl‐D‐erythritol phosphate (MEP) pathway of isoprenoid biosynthesis in bacteria. Source: [26]. This figure has been adapted from an open-source article distributed under the terms and conditions of the Creative Commons Attribution NonCommercial 4.0 International (CC BY-NC 4.0) license (https://creativecommons.org/licenses/by/4.0/).

In recent years, scientists have started to concentrate their study on potential strategies for eliminating bacteria that are resistant to antibiotics without necessarily developing new antibiotics. This strategy is intriguing when considering the expense and difficulties of finding and developing new antibiotics. Each kind of antibacterial antibiotic has a distinct mode of action that kills bacteria by attacking a particular region of their cells. It is also widely believed that the physiological effects of the antibiotics, such as loss of membrane permeability, alteration in the shape of the cell, or molecular events such as blockage of the key cellular pathways, affect how these diverse kinds of antibiotics function and different ways of killing bacteria [22]. After achieving their original targets, all antibiotic medications share a joint secondary impact, according to research by Calhoun et al. and Hong et al. The target bacterium is reportedly forced to develop "reactive oxygen species (ROS)," often referred to as free radicals, which, if not promptly neutralized, can seriously harm the DNA of bacteria and proteins [23,24]. This suggests that all bactericidal antibiotics, regardless of how they work, kill bacteria by having the same secondary impact on the bacterial cell. This notion was previously supported by research and discoveries by Kohanski et al., who showed that when tested against Gram-positive and Gram-negative bacteria, bactericidal drugs with different biological targets caused the creation of ROS. On the other hand, bacteriostatic antibiotics did not result in the generation of hydroxyl radicals; therefore, this was not the case [25]. Dual-acting immunoantibiotics, which target the non-mevalonate or methyl-D-erythritol phosphate pathway of isoprenoid biosynthesis and the riboflavin biosynthesis pathway in bacteria, have recently been presented (Figure 1) [26].

*A Crucial Stage in the Development of AR* *: The SOS Response*

In reply to DNA damage and oxidative stress, the emergency response is known as an inducible repair of the DNA pathway [27,28]. The growth of persister cells, stress resistance, and extended tolerance (including AR) are all facilitated by the Salt Overly Sensitive (SOS) pathway, which is crucial for adapting bacteria, pathogenicity, and diversification [29]. The SOS response was reportedly induced by antibiotic exposure, and mutants of bacteria that are not able to produce clusters of iron-sulfur became less vulnerable to bactericidal antibiotics, according to a study by Kohanski et al. [25]. This work also supports earlier findings that the SOS response can be induced by antibiotics like ciprofloxacin and that it may play a vital role in the emergence of drug resistance.

The Function of Hydrogen Sulfide (H2S) in Bacteria With Generalized AR

According to several studies, the synthesis of endogenous H2S by bacteria gives broad resistance to a diversity of antibiotics, leading to antibiotics or resistance in almost all the bacteria examined so far [30,31]. A novel resistance pattern mediated by H2S was first discovered by Shatalin et al. in various clinical isolates of *Escherichia coli,* *Pseudomonas aeruginosa*, *Bacillus anthracis*, and *Streptococcus aureus* [32]. According to some reports, exogenous H2S may not have the same antibacterial properties as endogenous H2S. Exogenous H2S is cytotoxic to various bacteria, including *Acinetobacter baumannii* and *Escherichia coli*, according to findings [33]. Many clinical uses for various H2S-releasing compounds have been developed recently, and their safety profiles have been described. Consideration of H2S-releasing chemicals becomes crucial to enhance antibiotic effectiveness and reverse AR in bacteria that do not produce H2S [34]. According to some reports, exogenous H2S may not have the same antibacterial properties as endogenous H2S. Exogenous H2S is cytotoxic to a number of bacteria, including *Escherichia coli* and *Acinetobacter baumannii*, according to research [35]. Similar findings were made by Podlesek and Bertok when researching the impact of exogenous H2S on the essential antimicrobial-resistant bacterium *Acinetobacter baumannii*, which lacks the genes necessary for H2S production. It was discovered that exogenous H2S was unsuccessful at shielding *Acinetobacter baumannii* from antibiotics. Antibiotics such as colistin, gentamycin, clarithromycin, and rifampicin, all of which are unrelated, were found to kill bacteria more effectively when H2S was present. However, it was also found that the bactericidal activity of the medications was significantly more vigorous when antibiotic-sensitive *Acinetobacter baumannii* were managed with H2S-releasing substances in addition to antibiotics than when the antibiotics were administered alone. Many different clinical applications of H2S-releasing molecules have been developed recently, and their safety profiles have been described. Consideration of H2S-releasing chemicals becomes crucial for enhancing antibiotic effectiveness and reversing AR in bacteria that cannot produce H2S [36].

Causes for concern

As is well known, H2S has both advantageous and detrimental effects on plants and animals. Surprisingly, both effects are related to energy metabolism and either prevention from or exacerbation of oxidative damage [37,38]. The third gasotransmitter in mammals like bears, after carbon monoxide and nitric oxide, has been found, and it has been linked to a number of metabolic activities [39,40]. Cysteine is degraded by the enzymes 3-mercaptopyruvate sulfurtransferase (3MST), cystathionine lyase (CSE), and cystathionine synthase (CBS) in humans and microorganisms [41]. This suggests that human cells depend on H2S, which is also produced by animal cells. In people, H2S acts as a signaling chemical, interacting with many organs, including the smooth muscle and brain. However, it is highlighted that the H2S concentration range is the main factor in determining the effect of H2S in an organism. Low levels of H2S are typically thought to be cytoprotective, whereas large doses (millimolar) are thought to be cytotoxic [42]. Bacterial cells in humans generate H2S via the CSE route. The tastes of bacterial and human CSE do, however, significantly vary. Bacterial CSE, 3MST, and CBS have been found to differ substantially from their mammalian equivalents, indicating the possibility of developing specialized inhibitors targeting these enzymes. Finding substances with a substantial affinity for the bacterial CSE will help to ensure that they are both specifically effective against bacteria and won't have any unintended negative impacts on the cells of mammals [43].

## Conclusions

AR is still a serious issue that needs urgent global attention. The idea of the "one compound, one target" approach, which has led to the development of antibacterial drugs, is effectively replaced by compounds or techniques that obstruct critical metabolic pathways. Although accommodating, the analytical trials and regulatory restrictions on this combinatorial approach to the discovery and advancement of antibiotics have their limitations. The development of antibiotics is a promising technique for inhibiting germs and enhancing the immune system. The various strategies that help in tackling AR have been implicated. Additionally, antibiotic-induced reduction of the bacteria's other repair mechanisms may be the best defense against bacterial infections.
